# Quantitative analysis of disfluency in children with autism spectrum disorder or language impairment

**DOI:** 10.1371/journal.pone.0173936

**Published:** 2017-03-15

**Authors:** Heather MacFarlane, Kyle Gorman, Rosemary Ingham, Alison Presmanes Hill, Katina Papadakis, Géza Kiss, Jan van Santen

**Affiliations:** 1 Center for Spoken Language Understanding, Institute on Development & Disability, Oregon Health & Science University, Portland, Oregon, United States of America; 2 Department of Pediatrics, Oregon Health & Science University, Portland, Oregon, United States of America; Waseda University, JAPAN

## Abstract

Deficits in social communication, particularly pragmatic language, are characteristic of individuals with autism spectrum disorder (ASD). Speech disfluencies may serve pragmatic functions such as cueing speaking problems. Previous studies have found that speakers with ASD differ from typically developing (TD) speakers in the types and patterns of disfluencies they produce, but fail to provide sufficiently detailed characterizations of the methods used to categorize and quantify disfluency, making cross-study comparison difficult. In this study we propose a simple schema for classifying major disfluency types, and use this schema in an exploratory analysis of differences in disfluency rates and patterns among children with ASD compared to TD and language impaired (SLI) groups. 115 children ages 4–8 participated in the study (ASD = 51; SLI = 20; TD = 44), completing a battery of experimental tasks and assessments. Measures of morphological and syntactic complexity, as well as word and disfluency counts, were derived from transcripts of the Autism Diagnostic Observation Schedule (ADOS). High inter-annotator agreement was obtained with the use of the proposed schema. Analyses showed ASD children produced a higher ratio of content to filler disfluencies than TD children. Relative frequencies of repetitions, revisions, and false starts did not differ significantly between groups. TD children also produced more cued disfluencies than ASD children.

## Introduction

Autism spectrum disorder (ASD) is characterized by deficits in communication, impairments in social interaction, and restricted or repetitive patterns of behavior, interests, and activities [1]. While linguistic abilities in children with ASD are highly variable [2, 3], delays and deficits are relatively common [4, 5]. Recent studies suggest a majority of verbally fluent children with ASD have impairments in structural language, which includes phonology, vocabulary, and grammar [6, 7]. On the other hand, pragmatic language—the socially-oriented elements of language use—is thought to be universally impaired in ASD [8–13]. Although many studies have attempted to measure specific features of pragmatic deficits in individuals with ASD and typically developing peers [14–18], pragmatic language has proved difficult to define and quantify [19, 20].

One area of particular pragmatic difficulty for individuals with ASD is conversational reciprocity. Children and adolescents with ASD experience difficulties with initiating conversation or responding to the initiations of others [18, 21, 22], taking conversational turns [23], staying on topic [24, 25], and producing coherent narratives [24]. These abilities are crucial for day-to-day speech communication, and thus there is great potential value for interventions that might increase the capacity of an individual with ASD to understand and be understood [26].

Disfluencies reflect difficulties in planning and delivering speech [27], and certain types of disfluency—particularly fillers like *uh* or *um*—make these difficulties explicit to listeners [28]. Disfluencies may also provide listeners with cues to linguistic structure, signal speaker uncertainty [29], or mark the introduction of new information to the discourse [30, 31]. Disfluencies, then, are also part of conversational reciprocity.

There is a clinical impression that individuals with autism “may lack in fluency” [9], and several exploratory studies attempted to quantify this impression. Studies investigating disfluency in individuals with ASD [32–34] have generally grouped disfluencies by function, under the hypothesis that different types of disfluency manifest from different types of processing breakdowns [35]. Previous studies have distinguished several major types of disfluency including pauses, fillers (*uh*, *um*), false starts, disfluent repetitions, revisions [36, 37], and stutters, a disruption in the expected rate or fluency of speech [17]. These studies have found that high-functioning adults with autism produce fewer revisions and more repetitions than typically developing controls [32]; children and adolescents with autism use more repetitions [38]; and children with autism produce longer silent pauses [39]. Another study found that children with ASD produced fewer “ungrammatical pauses” (pauses which occur within linguistic constituents like noun phrases; e.g., “the [pause] car”) while narrating a wordless picture book [40]. Finally, several studies have found that children with autism are less likely to produce *um*s than typically developing peers, but produce similar rates of *uh*s [39, 41, 42]. Thus, across different types of disfluencies and participant populations, there appear to be robust group differences. This is consistent with hypothesis that disfluencies reflect distinct types of processing breakdowns insofar as these breakdowns can be attributed to structural and pragmatic language difficulties associated with autism.

A significant challenge in interpreting these results—and specifically in relating them to known features of language in autism—is the lack of formal definitions of disfluency types like “false start” or “filler”. These interpretive challenges have lead some to argue for the utility of a single schema for coding disfluencies [43]. One well-known attempt to provide a comprehensive schema of disfluency proposes seven distinct types of disfluency plus one hybrid type [37] which can be classified using annotations of disfluencies and associated “repairs”. Though this schema provides formal definitions and many illustrative examples, it has not been widely adopted. In our opinion, the primary reason for this is that it is simply too complex, making it difficult to achieve high interannotator agreement under normal conditions. Thus, it is not apparent just what to count if one wishes to quantify patterns of disfluency use, an issue we seek to remedy.

### Current study

In what follows, we propose a precisely-defined schema for labeling disfluencies by type, and then apply this schema in a large-scale exploratory study of disfluency use in children with ASD, comparing them to peers with typical development (TD) or specific language impairment (SLI).

Specific language impairment is a neurodevelopmental disorder characterized by language delays or deficits in the absence of accompanying developmental or sensory impairments [44]. SLI is associated primarily with deficits in structural language abilities whereas ASD involves atypicalities in both structural and pragmatic language [45]. While some recent work has problematized the very term “specific language impairment” [46, 47], it is an appropriate label for a clinical group with intact non-verbal IQ and no other comorbidities, and this clinical group is essential to determining whether there are specific ASD-related profiles of disfluency use. For example, prior work has found that children with SLI produce more disfluencies than typically developing children matched on age, but similar rates to TD children matched on language [35, 48]. This suggests that difficulties with structural language—common in, but not specific to, children with ASD [49–51]—may affect disfluency rates. Without the SLI comparison group it would be impossible to isolate the influence of the pragmatic language difficulties which characterize ASD.

## Materials and methods

### Participants

115 children from the Portland, OR metropolitan area between 4–8 years of age participated in the current study: 51 children with autism spectrum disorder (ASD; 45 male), 44 children with typical development (TD; 32 male), and 20 children with specific language impairment (SLI; 12 male).

#### Recruitment and screening

As described in prior work [41], participants were recruited using a variety of community and health care resources. All participants scored 70 or higher for full-scale IQ using the Wechsler Preschool and Primary Scale of Intelligence (WPPSI-III; [52]) for children under 7 years of age, or the Wechsler Intelligence Scale for Children (WISC-IV; [53]) for children ages 7 or older. Children were excluded from the study if any of the following were present: 1) any other known metabolic, neurological, or genetic disorder, 2) gross sensory or motor impairment, 3) brain lesion, 4) orofacial abnormalities (e.g., cleft palate), or 5) intellectual disability. All participants were native, first-language speakers of English. During an initial screening, a certified speech-language pathologist confirmed the absence of speech intelligibility impairments and determined that the participants produced a mean length of utterance in morphemes (MLUM) of at least three.

#### Diagnostic groups

The gold standard for ASD diagnosis is best estimate clinical judgment (BEC) by experienced clinician diagnosis [54, 55]. In this study, a panel of two clinical psychologists, a speech language pathologist, and an occupational therapist, all of whom had clinical expertise with ASD, based their judgments on the DSM-IV-TR criteria for ASD [56]. The ASD group consisted only of children who received a consensus BEC diagnosis of ASD. The consensus diagnosis was further confirmed by above-threshold scores on two other tests: the Autism Diagnostic Observation Schedule-Generic (ADOS-G; [57]) according to the revised algorithm [58], and the Social Communication Questionnaire (SCQ; [59]) using a cutoff score of 12 as recommended for research purposes [60].

Language impairment was assessed using the Clinical Evaluation of Language Fundamentals (CELF), a test which produces a composite of expressive and receptive language abilities. The CELF Preschool-2 [61] was administered for children younger than 6 years of age; the CELF-4 [62] was used for children age 6 or older. Language impairment was determined when a participant received a CELF core language score (CLS) more than one standard deviation below the normative mean. Of the 51 children with ASD, this criterion identified 26 as language-impaired. Children in the SLI group also were required to have a documented history of language delays or deficits, and a BEC consensus judgment of language impairment (but not ASD). The clinical panel made this judgment using medical and family history, assessments performed as part of this study or at an earlier time by others, and school records. Children with a BEC diagnosis of SLI were excluded from the study if they reached threshold on both the ADOS-G and the SCQ.

Children who did not meet the above criteria for either ASD or SLI were assigned to the TD group. However, participants were excluded from the TD group (and the study) if they had any family members diagnosed with either ASD or SLI, a history of psychiatric disturbance (e.g. ADHD), or if they were above the aforementioned thresholds on either the ADOS-G or the SCQ.

### Procedures

Participants completed a battery of experimental tasks and cognitive, language, and neuropsychological assessments over six sessions of 2–3 hours each. Participating families were fully informed about study procedures and provided written consent. All experimental procedures were approved by the Oregon Health & Science University Institutional Review Board.

#### Standardized measures

Verbal IQ (VIQ), performance IQ (PIQ), and full-scale IQ (FSIQ) were estimated using the Wechsler scales tests, as described above.

The ADOS [57], a semi-structured autism diagnostic observation, was administered by an experienced clinician to all participants. Eleven children received ADOS Module 2, and 104 children received Module 3. The ADOS was scored according to the revised algorithms [58]. The social affect calibrated severity score (ADOS SA; range: 1–10) was calculated as clinician-reported measure of social communication difficulty. Transcripts of the ADOS were used to derive several other measures, as described below.

Parents completed the Behavior Rating Inventory of Executive Function (BRIEF) [63] for children 6 years of age or older, and the BRIEF-Preschool Version (BRIEF-P) [64] for children younger than 6 years. These were then used to compute the global executive composite (GEC), a measure of overall executive functioning.

The CELF core language score (CLS), and two CELF subscales, the expressive language index (ELI) and the receptive language index (RLI), were used to assess structural language abilities in children with ASD and SLI. Typically-developing children were screened for language impairment but did not complete the CELF.

Parents completed the Children’s Communication Checklist (CCC-2) [65], a 70-item questionnaire assessing the child’s communication abilities in natural settings. The general communication composite (GCC) is the sum of subscale scores from the eight CCC-2 domains related to communication (speech, syntax, semantics, coherence, initiation, scripted language, context, and nonverbal communication). The social-interaction deviance index (SIDI) then uses these subscales to measure relative strengths in structural versus pragmatic language. A negative SIDI indicates stronger relative structural language abilities while a positive score indicates stronger pragmatic language abilities.

Finally, parents completed the Social Communication Questionnaire [59], a 40-item assessment of symptomatology associated with ASD. The SCQ communication total score (SCQ-CTS; range: 0–12) [66] is the sum of scores for items in the communication domain and was also used as a parent-reported measure of communication abilities. The R package mice was used for the imputation of the SCQ data. Due to a small number of non-responses (<1%) to SCQ items, chained equation multiple imputation [67] was used to fill in non-responses before computing total scores.

An iterative procedure (implemented by the ldamatch R package) selected a subset of the sample consisting of the four groups (ALI: ASD with language impairment; ALN: ASD without language impairment; SLI; TD) according to the following constraints: 1) all four groups were matched on chronological age 2) SLI and ALI groups were matched on VIQ and PIQ, 3) ALN and TD groups were matched on VIQ and PIQ, and 4) ALI and ALN groups were matched on ADOS severity score. Groups were considered to match when the *P*-value for tests on the groups was ≥.2 for both a two-tailed Welch’s (unequal variance) *t*-test and an Anderson-Darling test [68].

#### Transcription

ADOS sessions were recorded and the child and examiner’s speech was transcribed verbatim using Praat software. Annotators were blind to participants’ diagnostic status and intellectual abilities. Transcriptions were generated in accordance with the Systematic Analysis of Language Transcripts (SALT) guidelines [69]. As per these guidelines, annotators were instructed to mark mazes (i.e., disfluent intervals of speech), including sequences of fillers and false starts, repetitions, and revisions. Annotators also segmented ADOS transcriptions into four activities: Play (including Make-Believe Play and Joint Interactive Play), Description of a Picture, Telling a Story from a Book (creating a story from a wordless picture book), and Conversation. For children who received the ADOS Module 2, the Conversation activity is any conversation that occurred outside all other structured activity; for children who received the ADOS Module 3, Conversation includes ADOS sections labeled Emotions, Social Difficulties and Annoyance, Friends and Marriage, and Loneliness, as well as any conversation that occurred outside all other structured activity. Other sections of the ADOS were not transcribed. Within each activity, annotators segmented conversational turns into individual utterances (or “C-units”), each consisting of (at most) a main clause and any subordinate clauses modifying it.

#### Measures derived from ADOS transcripts

ADOS transcripts were used to compute overall mean length of utterance in morphemes (MLUM) [70] using SALT software [69]. MLUM is a simple, face-valid measure of morphological and syntactic complexity recommended for measuring spoken language development in children with autism [71]. ADOS transcripts were also used to count number of utterances, fluent words (words which are not part of a maze), and disfluent intervals for each participant.

#### Schema for disfluency coding

We propose a disfluency schema which simplifies the eight disfluency types proposed in prior work [37] by grouping them into a smaller set of four major types. In what follows we use the term *disfluent interval* to refer to one or more mazes, optionally followed by a related “repair”. We use the term *content maze* to refer to disfluencies which contain content words (in contrast to fillers such as *uh* or *um*). During the transcription process, annotators indicated mazes with parentheses and any associated repairs with curly braces. For example, in the utterance *“I like going to the (pool) {park}”*, the maze is *(pool)* and the repair is *{park}*. A disfluent interval may consist of multiple types of disfluencies; for example, in the utterance *“I like going to the (pool) (um) {park}”*, the disfluent interval contains a content maze, a filler *um*, and a repair.

Our schema categorizes disfluencies according to a small number of broad functional types. We distinguish four types of “repair”. In a *repetition*, the maze and repair are identical. When the maze and repair are not identical, the disfluent interval is classified as a *revision*. Revisions are thus disfluencies where the speech is “edited” in some fashion. Within *revision*, it is possible to discern two subtypes—not analyzed separately in this study—which we call *deletion* and *insertion*. In these disfluency types the repair can be formed strictly by deletion of word(s) present in the maze, or insertion of word(s) not present in the maze, respectively. *False starts* are content mazes which lack a corresponding repair. *Fillers* consist of a fixed set of “filled pauses” such as *um* and discourse markers such as *you know*. Mazes that are less than a single prosodic word (i.e., a “stutter”) are ignored. These types are exemplified in Table 1.

**Table 1:** Disfluency type schema.

**Repetition—REP**	(y){y}z	*(My) {My} dog is nice*.
	mid-word interruption	*My (do-) {dog} is nice*.
**Revision—REV**	(xy){xz}	*(My dog) {My cat} is nice*.
		*She (goes) {went}*.
	(xz){yz}	*(He’s very) {She’s very} friendly*.
[Deletion—DEL]	(xyz){xz}	*(My old dog) {My dog} is nice*.
	(xy){x}	*My dog (likes to) {likes} food*.
[Insertion—INS]	(xz){xyz}	*(My dog) {My yellow dog} is nice*.
	(y){xy}	*My dog likes (the) {all the} food*.
**False start—FS**	(xyz) abc	*(But what if) My friend likes dogs*.
**Filler—F**	discourse markers and filled pauses	*like*, *um*, *uh*, *mm*, *hmm*, *I mean*, *ah*

Finally, we consider a maze to have been *cued* when a filler occurs between the maze and repair portions of a repetition or revision, or when a filler occurs immediately before or after any type of content maze, as in the revisions *“(cat) (um) {dog}”* or *“(um) (cat) {dog}”*.

All complete utterances containing at least one maze (denoted by parentheses in the original transcription) were then annotated with curly braces inserted to indicate the span of any repair. The annotator who marked these repair intervals was an experienced transcriber who had not participated in the initial SALT annotation efforts. The annotator was permitted to modify disfluency boundaries when they felt the maze was incorrectly delineated by the original transcriber, though this was rarely necessary.

A computer program (S1 and S2 Files) was then used to group mazes and repairs into disfluent intervals and to categorize each disfluent interval according to the schema given in Table 1. This program was subject to extensive unit testing to verify its correctness, and is included as Supporting Information. Raw counts of each disfluency type were determined automatically by this computer program and then computed for each child and activity.

This two-part process begins with manual annotations and concludes with automated categorization, though some recent work has suggested that the annotation step can be automated with the help of natural language processing techniques [72, 73]. However, we note that both annotation and categorization could just as well have been performed manually.

### Statistical analysis

Inferential analyses were conducted to detect group differences in the use of fillers and content mazes. In preliminary analyses, it was determined that the counts of individual disfluent events, pooled across participants, failed to satisfy the statistical independence assumptions of logistic regression. Therefore, inferential analyses were conducted using mixed effects logistic regression [74] with a per-subject random intercept. The R package lme4 was used for mixed effects regressions and multcomp for the post hoc tests. The primary independent variable was participant group (ASD, SLI, or TD). All models also included one subject-linked covariate, verbal IQ. Each token was coded for ADOS activity (Play, Description of a Picture, Telling a Story from a Book, or Conversation). To facilitate interpretation, continuous variables were z-transformed, and sum coding was used to encode categorical variables. The likelihood ratio test was used to test for significance of individual predictors, and the Tukey HSD test was used to test for significant differences within factor groups [75]. Exploratory analyses were conducted by measuring correlations with Kendall’s *τ*_*b*_, a non-parametric correlation statistic.

To confirm that our results are in no way influenced by combining data from children who completed both modules, we repeated all statistical analyses excluding data from children who completed the Module 2 and obtained the same main effects as with the combined data. As a result, we report the full analyses with both modules.

## Results

The matching procedure retained 97 of the 115 children (ASD: 47, SLI: 18, TD: 32). Summary statistics for the resulting age-matched sample are reported in Table 2.

**Table 2:** Group summary statistics.

	ASD (*n* = 47)		SLI (*n* = 18)		TD (*n* = 32)		
	mean	(s.d.)	mean	(s.d.)	mean	(s.d.)	*P*_HSD_ < .05
CA	6.7	(1.1)	7.1	(1.0)	6.8	(1.0)	(none)
FSIQ	98.0	(15.8)	88.7	(8.0)	117.7	(11.3)	SLI < ASD < TD
VIQ	94.8	(17.8)	86.1	(6.1)	116.9	(12.9)	SLI = ASD < TD
PIQ	108.6	(17.8)	101.7	(12.3)	117.6	(13.2)	SLI = ASD < TD
GEC	68.7	(8.8)	65.8	(13.0)	44.6	(8.1)	TD < SLI = ASD
CLS	88.9	(21.8)	73.9	(8.2)	n.a.	(n.a.)	SLI < ASD
MLUM	4.2	(1.0)	4.1	(1.0)	5.1	(1.0)	SLI = ASD < TD
GCC	50.9	(11.0)	48.2	(12.4)	95.7	(12.9)	SLI = ASD < TD
SCQ	19.7	(4.9)	11.2	(6.5)	2.7	(2.2)	TD < SLI < ASD
ADOS	7.6	(1.9)	2.9	(2.7)	1.2	(0.5)	TD < SLI < ASD

Mean and standard deviation for each group, and post hoc group contrasts which are significant at *α* = .05. CA = chronological age in years; FSIQ = full-scale IQ; VIQ = verbal IQ; PIQ = performance IQ; GEC = BRIEF global executive composite; CLS = CELF core language score (not available for TD); MLUM = mean length of utterance in morphemes; GCC = CCC-2 general communication composite; SCQ = SCQ total score; ADOS = ADOS-G calibrated severity score.

Table 3 shows counts of each disfluency type for each group.

**Table 3:** Number of disfluencies by type and group.

	ASD (*n* = 47)	SLI (*n* = 18)	TD (*n* = 32)
False start	1,180	410	754
Filler	1,269	588	1,867
Revision	695	202	503
Repetition	1,315	352	659
Total	4,459	1,552	3,783

The first goal of this study was to develop and evaluate an automated system of disfluency detection. To evaluate the manual coding done on the original utterances, we examined inter-annotator agreement. This was assessed by drawing a stratified random sample (consisting of four disfluent utterances from each child) which was then coded by a second annotator using the same guidelines. If there was more than one maze in an utterance, only the first was used to test agreement. Two forms of agreement were measured: agreement on identification of repair spans, and agreement on disfluency types assigned to each maze. Both annotators marked the same repair span in 90% of the cases. Whether or not both annotators marked the same repair span, the computer program assigned the same disfluency type for the disfluent interval in 91% of the cases. Cohen’s kappa (*κ*) for computer-annotator agreement in disfluency types was.904, corresponding to “almost perfect” agreement according to the Landis and Koch [76] qualitative guidelines.

### Frequency of fillers versus content mazes

The first inferential analysis tested for group differences in the relative frequencies of fillers (such as *um*, *uh*, and *like*) versus content mazes; the results are shown in Table 4.

**Table 4:** Results for regression on content mazes versus fillers.

	Log-odds	S.E.s	*χ*^2^	*P*(*χ*^2^)
(Intercept)	0.670	0.09		
Group:			12.18	.002
ASD	0.386	0.11		
SLI	−0.055	0.16		
TD	−0.331	0.15		
VIQ	−0.001	0.09	0.00	.988
ADOS Activity:			120.34	<.001
Play	0.074	0.04		
Description Of A Picture	−0.088	0.04		
Telling A Story From A Book	0.360	0.05		
Conversation	−0.347	0.03		

Mixed effects logistic regression on the relative frequencies of content mazes versus fillers; predictors which favor content mazes have positive log-odds and predictors which favor fillers have negative log-odds. VIQ = verbal IQ.

**Fig 1:**
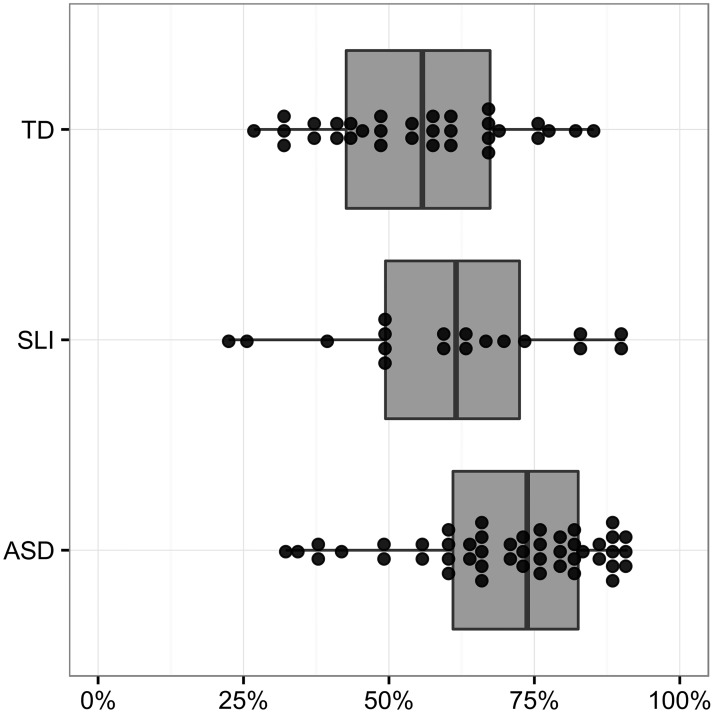
Percent of mazes which are content mazes (versus fillers).

There was a significant effect of group (*χ*^2^ = 12.18, *d*.*f*. = 2, *P* <.002). A post hoc test revealed a significant difference between the ASD and TD groups (*P* = .010); in the ASD group 72% of disfluencies were content mazes, whereas in the TD group only 51% of disfluencies were content mazes. Fig 1 illustrates these percentages, with each dot representing a participant’s percentage of content disfluencies (versus content disfluencies and fillers combined). The SLI group produced a content to filler ratio between that of the other two groups, but was not significantly different from either of these other groups.

There was also a main effect of ADOS activity (*χ*^2^ = 120.34, *d*.*f*. = 3, *P* <.001). Telling A Story From A Book most favored the use of content mazes, followed by Play, Description Of A Picture, and Conversation. In post hoc tests, all pairs of activities were significantly different from other pairs of activities (all *P* <.001, except for Play and Description of a Picture, for which *P* = .004).

### Frequency of content maze types

The second set of inferential analyses tested for group differences between the three major classes of content mazes: repetitions, revisions (including insertions and deletions), and false starts. These analyses were performed using two regressions: one comparing the relative frequencies of repetitions versus revisions, and another comparing false starts versus repetitions and revisions.

**Fig 2:**
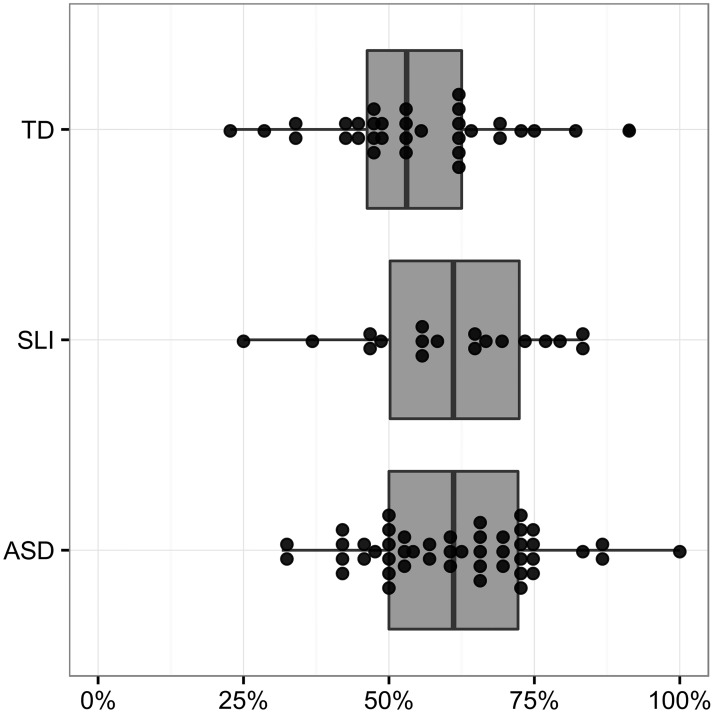
Percent of content mazes which are repetitions (versus revisions).

The results for the regression on repetitions versus revisions are shown in Table 5. There were no significant main effects in this regression. Fig 2 illustrates these percentages, with each dot representing a participant’s percentage of repetitions (versus repetitions and revisions combined).

**Table 5:** Results for regression on repetitions versus revisions.

	Log-odds	S.E.s	*χ*^2^	*P*(*χ*^2^)
(Intercept)	0.380	0.07		
Group:			1.442	.486
ASD	0.090	0.08		
SLI	0.007	0.12		
TD	−0.097	0.11		
VIQ	−0.075	0.06	1.49	.222
ADOS Activity:			2.45	.493
Play	0.023	0.06		
Description Of A Picture	0.025	0.07		
Telling A Story From A Book	−0.108	0.08		
Conversation	0.060	0.06		

Mixed effects logistic regression on the relative frequencies of repetitions versus revisions; predictors which favor repetitions have positive log-odds and predictors which favor revisions have negative log-odds. VIQ = verbal IQ.

**Fig 3:**
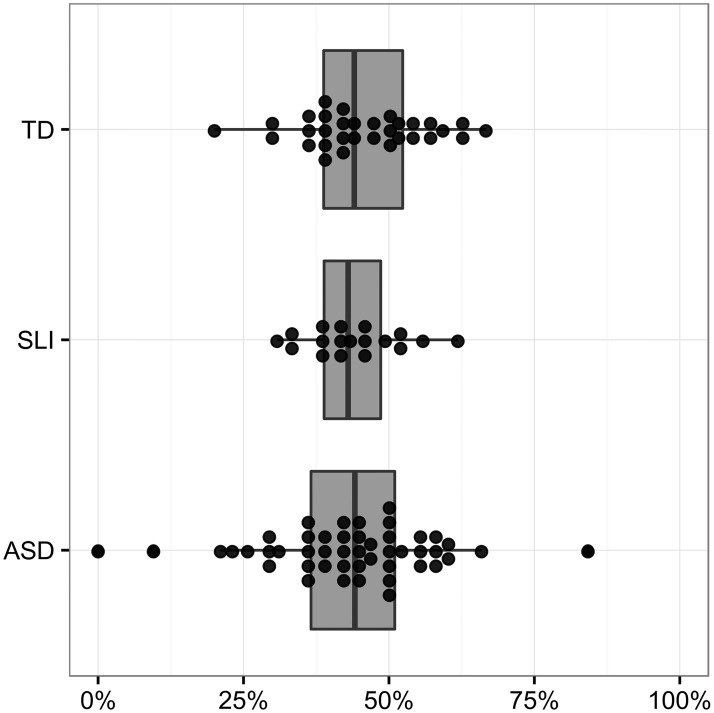
Percent of content mazes which are false starts (versus repetitions and revisions).

The results of the regression on false starts versus repetitions and revisions are shown in Table 6. There were no significant main effects. Fig 3 illustrates these percentages, with each dot representing a participant’s percentage of false starts (versus repetitions and revisions).

**Table 6:** Results for regression on false starts versus repetitions and revisions.

	Log-odds	S.E.s	*χ*^2^	*P*(*χ*^2^)
(Intercept)	−0.188	0.05		
Group:			2.27	.322
ASD	0.000	0.06		
SLI	0.117	0.09		
TD	−0.118	0.08		
VIQ	0.115	0.04	6.63	.010
ADOS Activity:			7.91	.049
Play	−0.078	0.05		
Description Of A Picture	0.112	0.05		
Telling A Story From A Book	−0.067	0.06		
Conversation	0.033	0.04		

Mixed effects logistic regression on the relative frequencies of the use of false starts versus repetitions and revisions; predictors which favor false starts have positive log-odds and predictors which favor repetitions or revisions have negative log-odds. VIQ = verbal IQ.

### Use of fillers as cues

The final inferential analysis tested for group differences in the cueing of content mazes (as defined above); the results are shown in Table 7.

**Table 7:** Results for regression on cued versus non-cued content mazes.

	Log-odds	S.E.s	*χ*^2^	*P*(*χ*^2^)
(Intercept)	−0.852			
Group:			7.22	.027
ASD	−0.194	0.07		
SLI	0.032	0.10		
TD	0.162	0.10		
VIQ	0.062	0.06	1.24	.265
ADOS Activity:			45.65	<.001
Play	−0.071	0.04		
Description Of A Picture	0.061	0.03		
Telling A Story From A Book	−0.164	0.04		
Conversation	0.174	0.03		

Mixed effects logistic regression on relative frequencies of the use of cued versus non-cued content mazes; predictors which favor cued content mazes have positive log-odds and predictors which favor non-cued content mazes have negative log-odds. VIQ = Verbal IQ.

**Fig 4:**
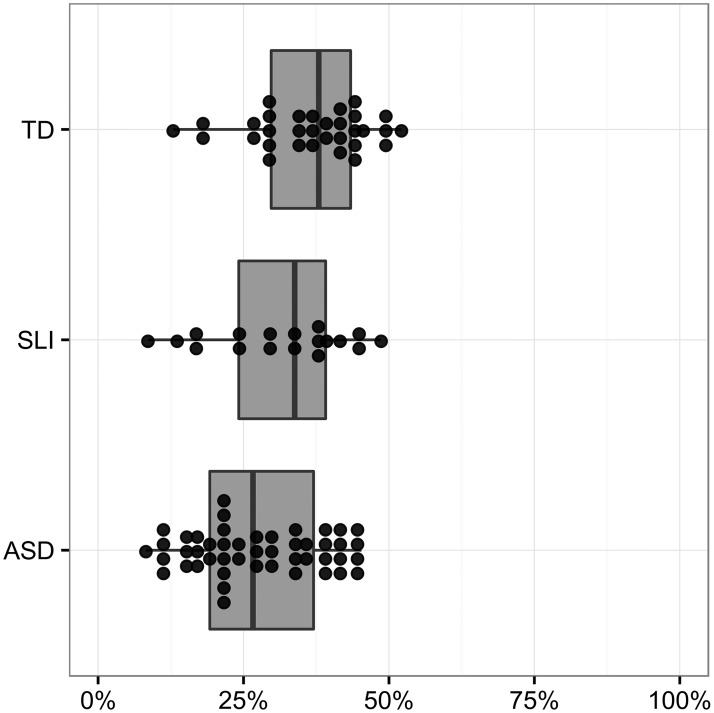
Percent of content mazes which are cued by a filler (versus content mazes which are not cued).

There was a main effect of group (*χ*^2^ = 7.22, *d*.*f*. = 2, *P* = .027). Post hoc tests revealed a marginal difference between the ASD and TD groups (*P* = .085); 29% of content mazes were cued in the ASD group whereas 41% were cued in the TD group. The SLI group produced cued content mazes at a rate between that of the other two groups, but was not significantly different from either of these other groups. Fig 4 illustrates these percentages, with each dot representing a participant’s percentage of cued content mazes (versus uncued content mazes).

There was also a main effect of activity (*χ*^2^ = 45.65; *d*.*f*. = 3; *P* <.001). In post hoc tests, Conversation favored cued content mazes more than Play and Telling A Story From A Book (both *P* <.001); Picture Description also favored cued mazes significantly more than did Telling a Story From a Book (*P* = .008).

### Exploratory analysis

Exploratory analyses were conducted to investigate whether the two dependent variables correlated with group might also be modulated by within-group heterogeneity. Within-group independent variables included measures of age, intellectual ability, executive function, structural and pragmatic language, and social communication. Within each group, and for each dependent and independent variable, the Kendall *τ*_*b*_ coefficient was computed and the resulting *P*-values adjusted so as to control for within-group false discovery rate [77]. Many of these tests are complementary—i.e., several pairs of independent variables are highly correlated and measure closely-related constructs—and the resulting statistical tests are very likely underpowered (particularly in the SLI group), so the resulting *P*-values should be interpreted with caution.

The first exploratory analysis targeted content maze versus filler use. As shown in Table 8, no within-group effect reached significance.

**Table 8:** Results for exploratory analysis of content versus non-content mazes.

	ASD	SLI	TD
CA	.02	.10	−.31
FSIQ	−.05	.17	.15
VIQ	−.08	−.15	.11
PIQ	.00	.17	.18
GEC	.02	.17	.03
CELF			
CLS	−.13	−.06	n.a.
ELI	−.14	−.01	n.a.
RLI	−.13	.13	n.a.
MLUM	.11	.23	−.04
CCC-2			
GCC	−.04	−.10	−.04
SIDI	−.02	−.12	−.24
SCQ COM	.10	.40	−.13
ADOS SA CSS	−.19	.02	.01

Associations between per-child content mazes (versus filler) use and age, intellectual ability, executive function, language, and social ability, as measured by Kendall’s *τ*_*b*_. Children in the TD group did not complete the CELF. CA = chronological age in years; FSIQ = full-scale IQ; VIQ = verbal IQ; PIQ = performance IQ; GEC = BRIEF global executive composite; CLS = CELF core language score; ELI = CELF expressive language index; RLI = CELF receptive language index; MLUM = mean length of utterance in morphemes; GCC = CCC-2 general communication composite; SIDI = CCC-2 social-interaction deviance index; SCQ COM = SCQ communication total score [66]; ADOS SA CSS = ADOS-G social affect calibrated severity score; there were no statistically significant differences at *P* = .05 after correction for false discovery rate [77].

The second regression targeted cued versus non-cued content mazes; the results are shown in Table 9. Three tests were significant after correcting for false discovery rate. Within the ASD group, verbal IQ and the CELF core language score, expressive language index, and receptive language index were positively correlated with cueing of content mazes. Within the TD group, chronological age was positively correlated with cueing of content mazes.

**Table 9:** Results for exploratory analysis of cued (versus non-cued) content mazes.

	ASD	SLI	TD
CA	.06	.11	.47*
FSIQ	.22	−.12	−.10
VIQ	.28*	.28	−.08
PIQ	.07	−.17	−.05
GEC	.09	−.13	.01
CELF			
CLS	.33*	.01	n.a.
ELI	.34*	−.07	n.a.
RLI	.28*	.07	n.a.
MLUM	.10	−.04	.21
CCC-2			
GCC	.02	.11	−.01
SIDI	−.16	.05	.26
SCQ COM	−.06	−.27	.11
ADOS SA CSS	.04	−.08	−.20

Associations between per-child cued (versus non-cued) content maze use and age, intellectual ability, executive function, language, and social ability, as measured by Kendall’s *τ*_*b*_. Children in the TD group did not complete the CELF. CA = chronological age in years; FSIQ = full-scale IQ; VIQ = verbal IQ; PIQ = performance IQ; GEC = BRIEF global executive composite; CLS = CELF core language score; ELI = CELF expressive language index; RLI = CELF receptive language index; MLUM = mean length of utterance in morphemes; GCC = CCC-2 general communication composite; SIDI = CCC-2 social-interaction deviance index; SCQ COM = SCQ; ADOS SA CSS = ADOS-G social affect calibrated severity score; * = statistically significant at *P* = .05 after correction for false discovery rate [77].

## Discussion

In this paper we first proposed a simple schema for categorizing speech disfluencies by type. Compared to the methods of prior studies of disfluency in autistic populations, this schema is more thoroughly specified and exemplified, but also far less complicated than the most expressive schemata [37]. This system can be applied automatically or manually, and allows us to achieve excellent inter-annotator agreement.

We note that while this schema for disfluency coding was developed specifically for this study, it was completed before any inferential analyses were performed, yet we find substantial group differences largely consistent with prior work. This suggests that it has potential utility in the study of disfluency more generally. For instance, it might be used in research on automating maze detection [72, 73, 78] for computer-aided language sample analysis.

We used this schema for coding disfluency type in a large corpus consisting of speech from children with autism, specific language impairment, and typical development. We found that, on average, the ASD group strongly favored content mazes over fillers, whereas the TD group produced roughly the same number of content mazes and fillers. This result is largely consistent with a prior study of adults with ASD [32] reporting that adults with ASD produced fewer fillers and more disfluent repetitions than TD peers. However, in contrast to prior studies [32, 38] we found no group differences in the usage frequency of the three types of content mazes.

We also found a group difference in the use of filler “cues” to content mazes. To understand a disfluent utterance like *“I like going to the (pool) {park}”*, listeners must mentally excise the maze *(pool)* and replace it with the repair *{park}*. One study argues that “cues” to disfluent speech—defined as fillers, explicit editing terms, or long pauses—may aid listeners in this process [79], and found that speakers are better able to identify speech as disfluent when the disfluent interval is cued. In this study, we found that filler cues to content mazes were significantly less common in the ASD group than the TD group. As the absence of these cues may make it more difficult to be understood, we hypothesize that this may contribute to the conversational reciprocity difficulties associated with ASD.

However, it is also possible that this effect is caused by group differences in other social-cognitive abilities. For example, executive functioning difficulties are associated with higher rates of disfluency in typical adolescents and adults [80], suggesting that disfluencies reflect difficulties in planning and delivering speech [27]. Under the hypothesis that filler cues are intended to help facilitate understanding, the additional planning required to produce a filler cue may be more difficult for children with ASD, as executive functioning difficulties are more common in individuals with ASD [81]. Another possibility is that general developmental maturity is a factor, given the significant positive correlation between content maze cueing and chronological age.

We used a group of children with specific language impairment as a comparison group to help isolate the effects of structural language deficits, characteristic of children with SLI though also common in children with ASD, from pragmatic language deficits, characteristic of children with ASD. While we found significant group effects for two variables (the relative frequency of fillers vs. content mazes, and the use of filler cues with content mazes), the SLI group fell between the ASD and TD groups for both variables, and post-hoc contrasts were non-significant. Thus despite our care, our findings concerning the relative role of structural vs. pragmatic language abilities are somewhat inconclusive.

We hypothesized that different ADOS activities might influence disfluency use, and in this study ADOS activity emerged as one of the most robust predictors of disfluency use, further highlighting the importance of controlling for topic in quantitative studies of pragmatic language. Across diagnostic groups, the ADOS activity “Telling A Story From A Book” accounted for the highest rate of content mazes while the ADOS activity “Conversation” accounted for the highest rate of cued content mazes.

An anonymous reviewer asks whether the disfluencies studied here might be related to apraxia. This issue is addressed directly in a recent study [82] which measured a number of features of speech, prosody, and voice quality using spontaneous speech samples from children with ASD, childhood apraxia of speech (CAS), speech delay, or typical development; inclusion criteria for the ASD and TD groups, as well as elicitation procedures, were quite similar to the current study. The authors found only one feature, “Increased Repetitions and Revisions”, for which the ASD and CAS groups were not significantly dissimilar. While the current study also finds elevated rates of content mazes—of which repetitions and revisions are the most common types—in children with ASD, we are reluctant to interpret our findings as support for the hypothesis that apraxia is a causal factor in atypical speech in children with ASD.

This study had several limitations. First, participants were drawn from a relatively wide age range (4–8). Though chronological age was included as a covariate in regression analyses, developmental differences may have obscured important group differences. Secondly, the majority of the participants were male. Consequently, we lacked statistical power to investigate gender differences. Furthermore, we did not investigate the role of socioeconomic status, though it may play a role in expressive language abilities [83, 84] including use of disfluency [85, 86]. Another limitation is that the diagnostic groups were defined using strict cutoffs for specific language impairment and ASD; different cutoffs might produce different results. All participants were high-functioning, limiting the generalizability of these results to the larger population of individuals with ASD. Finally, disfluency was coded using a set of formal but relatively coarse categorical types; a more granular classification of disfluencies applied to an even larger sample might produce different results.

The current study was limited to disfluency as it is expressed in English. However, the general patterns documented here are not necessarily limited to children acquiring English; indeed, content mazes and fillers appear to be a linguistic universal [87]. Thus, it is possible that similar patterns will be found in children acquiring other languages. We leave this as a topic for future research.

Our analyses uncovered substantial differences in disfluency use between children with and without ASD. Given that social-communicative deficits are a defining feature of ASD, these differences provide convergent evidence for the listener-oriented function of disfluencies in the speech of typical individuals [27, 88, 89]. Furthermore, if group differences in use of cued mazes are replicated, then this, along with other subtle aspects of pragmatic language, may be a useful target for intervention in individuals with ASD who are verbal and high-functioning.

## Conclusions

This study investigated disfluent speech in autism, using groups with specific language impairment and typical development as controls so as to provide a concrete quantitative characterization of a clinical impression. We proposed a simple schema for coding disfluency type, applicable to studies of disfluency more generally, and using this schema, we found that children with ASD have different patterns of disfluency than peers with SLI or typical development, including a higher rate of content maze use and a lower rate of filler use. The patterns of disfluency investigated here are easily quantified features of pragmatic language that may differentiate ASD and SLI, a challenging differential diagnosis [90–92]. We recommend that future studies quantify disfluency patterns in longitudinal studies of the same populations, similar to a recent longitudinal study of disfluency in typically developing children [93].

## Supporting information

S1 File Python library for computing a minimum-edit distance alignment between two strings. (PY)

S2 File Python library for coding mazes according to the disfluency schema. (PY)
